# Assessing technical and biological variation in SWATH-MS-based proteomic analysis of chronic lymphocytic leukaemia cells

**DOI:** 10.1038/s41598-021-82609-2

**Published:** 2021-02-03

**Authors:** Gina L. Eagle, John M. J. Herbert, Jianguo Zhuang, Melanie Oates, Umair T. Khan, Neil R. Kitteringham, Kim Clarke, B. Kevin Park, Andrew R. Pettitt, Rosalind E. Jenkins, Francesco Falciani

**Affiliations:** 1 Department of Molecular and Clinical Cancer Medicine, Institute of Systems, Molecular and Integrative Biology, University of Liverpool, Liverpool, UK; 2 Computational Biology Facility, University of Liverpool, Liverpool, UK; 3 Department of Haemato-Oncology, Clatterbridge Cancer Centre NHS Foundation Trust, Liverpool, UK; 4 Department Pharmacology and Therapeutics, Institute of Systems, Molecular and Integrative Biology, MRC Centre for Drug Safety Science, University of Liverpool, Liverpool, UK; 5 Department of Biochemistry and Systems Biology, Institute of Systems, Molecular and Integrative Biology, University of Liverpool, Liverpool, L69 7ZB UK

**Keywords:** Biological techniques, Computational biology and bioinformatics, Biomarkers, Oncology

## Abstract

Chronic lymphocytic leukaemia (CLL) exhibits variable clinical course and response to therapy, but the molecular basis of this variability remains incompletely understood. Data independent acquisition (DIA)-MS technologies, such as SWATH (Sequential Windowed Acquisition of all THeoretical fragments), provide an opportunity to study the pathophysiology of CLL at the proteome level. Here, a CLL-specific spectral library (7736 proteins) is described alongside an analysis of sample replication and data handling requirements for quantitative SWATH-MS analysis of clinical samples. The analysis was performed on 6 CLL samples, incorporating biological (IGHV mutational status), sample preparation and MS technical replicates. Quantitative information was obtained for 5169 proteins across 54 SWATH-MS acquisitions: the sources of variation and different computational approaches for batch correction were assessed. Functional enrichment analysis of proteins associated with IGHV mutational status showed significant overlap with previous studies based on gene expression profiling. Finally, an approach to perform statistical power analysis in proteomics studies was implemented. This study provides a valuable resource for researchers working on the proteomics of CLL. It also establishes a sound framework for the design of sufficiently powered clinical proteomics studies. Indeed, this study shows that it is possible to derive biologically plausible hypotheses from a relatively small dataset.

## Introduction

Chronic lymphocytic leukaemia (CLL) is the most common leukaemia in adults in Western countries. It is a malignancy of CD5^+^ B lymphocytes that accumulate in the blood, bone marrow and secondary lymphoid tissues such as lymph nodes^1^. CLL is a highly heterogeneous disease and is characterised by its clinical variability, particularly in relation to treatment response^2^. This clinical variability is partially reflected by two distinct forms of the disease defined by the somatic mutational status of the immunoglobulin heavy chain variable region (IGHV) gene. Thus, patients whose CLL cells express mutated IGHV genes (M-CLL) are associated with a favourable outcome whereas those with CLL cells expressing unmutated IGHV genes (UM-CLL) are associated with early disease progression and shorter survival^3–5^. In addition, many other factors are also thought to be associated with this clinical variability; they include distinct pattern of clonal evolution and reciprocal interactions between leukemic cells and the tissue microenvironment resulting in the activation of pro-survival signalling pathways^1^. Indeed, the B-cell receptor (BCR) signalling pathway is critically involved in the survival and proliferation of CLL cells^2^.

Past attempts at understanding the biological basis of the heterogeneity between CLL patients have mainly focussed on genomic alterations and gene expression at the mRNA level^6^. However, despite this, the molecular basis of CLL variability remains incompletely understood. We speculate that the in-depth study of the CLL proteome could thus provide better understanding of CLL heterogeneity and its underlying biological mechanisms. There are a limited number of studies that have applied proteomic approaches to link individual protein expression to the clinical phenotype in CLL^7–11^. However, large-scale CLL proteomic studies are still lacking^12^. Mass spectrometry (MS) is the standard method of choice for measuring protein expression^13^, with shotgun MS using data dependent acquisition (DDA) being the dominant approach in cancer proteomics research to date^14^. However, fast, reproducible and sensitive detection and quantification of proteomes in a large number of patient samples has remained a challenge due to limitations in technology. Recently, data independent acquisition (DIA) technologies have emerged as an alternative to DDA.

SWATH (Sequential Windowed Acquisition of all THeoretical fragments)-MS, is a label-free mass spectrometric technique that combines DIA with targeted data extraction on a high-resolution mass spectrometer^15^. SWATH-MS generates mass spectral maps of fragment ions from all detectable peptide precursors. The composite MS/MS spectra are then deconvoluted by alignment with a high quality and comprehensive tissue-specific library^16^, whereupon patient samples can be stratified based on the quantitative expression profile of thousands of proteins. SWATH-MS has been shown to be a highly reproducible method for large-scale protein quantification^17^. However, a comprehensive analysis of the sources of variation associated with large-scale sample preparation and instrument robustness is still lacking. Such analyses are needed for the optimisation of experimental and data workflows for optimal study design and, ultimately for routine, high-throughput clinical proteomics. In addition, due to the heterogeneous nature of CLL, biological variability between patient samples has to be considered to ensure that sufficient numbers of samples are included in a SWATH-MS study for robust statistical discrimination between clinical subgroups.

In this study, SWATH-MS for the proteome-wide analysis of CLL patient samples was optimised. To achieve this, a comprehensive CLL-specific spectral library was generated. SWATH-MS data was then acquired from cryopreserved CLL samples from 6 patients at various stages of the disease, incorporating triplicate sample preparations and triplicate MS acquisitions for each sample into the experimental design. The relative contribution of the technical variability, naturally associated with sample handling and with the acquisition technology, and biological variability (IGHV mutational status) in the generation of SWATH-MS data was then assessed. A robust statistical approach to correct for technical variations was applied and analysis of the proteins found to be differentially expressed between UM-CLL and M-CLL was performed. Pathway analysis performed on these proteins supported the importance of metabolic remodelling in the biology of CLL and remarkably gene set enrichment analysis showed considerable overlap with previous studies based on gene expression profiling. Finally, an error model to estimate the statistical power of a SWATH-MS study was developed. This model determined the numbers of CLL samples required to detect significant changes in protein expression across the whole dynamic range of a SWATH-MS dataset.

Our study highlights the importance of assessing biological and technical variability in SWATH-MS generated protein expression data prior to undertaking large-scale clinical proteomic studies.

## Results

### Generation of a CLL-specific spectral library for SWATH-MS analysis

A CLL-specific spectral library to support quantitative proteomics of CLL samples by SWATH-MS has been generated. The library contains 1,586,900 spectra (< 1% FDR) and digital information for 157,285 peptides (< 1% FDR) resulting in the identification of 7736 proteins (the full list of proteins is provided in the DDA “Supplementary data”).

**Figure 1:**
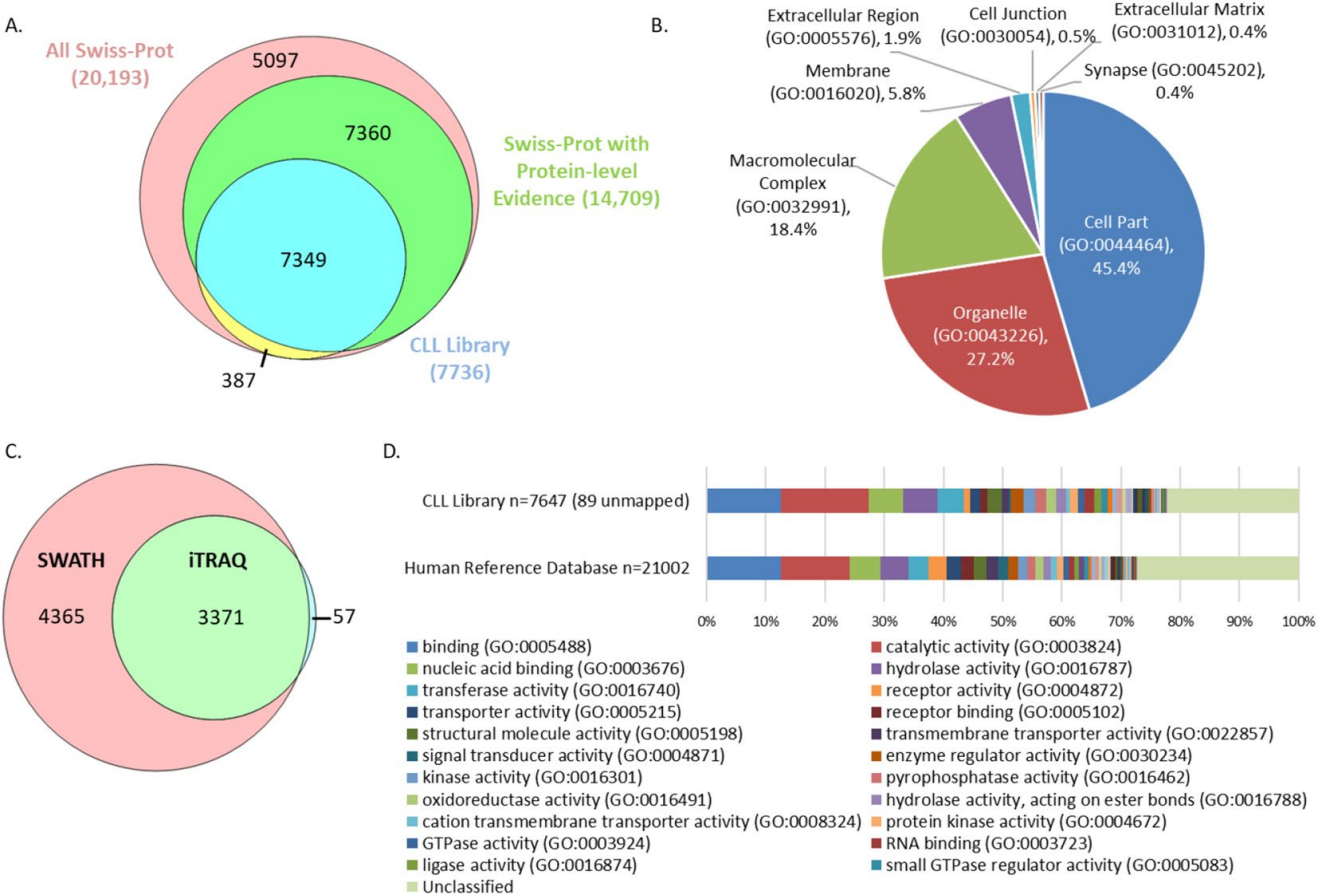
Representation of proteins in CLL-specific spectral library. (**A**) Representation of proteins from the CLL library (n = 7736) in the UniProtKB/Swiss-Prot Human database, showing coverage of 50% of entries with evidence at the protein level. (**B**) GO cellular components of proteins represented in the CLL library showing the known cellular location for 5499 entries. (**C**) Overlap of proteins identified in our previous iTRAQ study^7^ and represented in the CLL library. The CLL library includes 99% of the proteins identified in our previous study and expands coverage of the CLL proteome by 127%. (**D**) Proteins represented in the CLL library with known GO Molecular Functions (n = 7647) compared with representation from entries in the reference Homo sapiens gene database (n = 21,002).

The library encompasses 50% of all human UniProtKB/SwissProt entries that have evidence at the protein level (Fig. 1A) and represents a broad range of Gene Ontology (GO) cellular components (PANTHER) (Fig. 1B). The library covers 98% of the CLL proteome previously reported in our iTRAQ-MS study^7^ and expands the coverage by 127% (Fig. 1C). The quality of the library and its potential as a reference for future functional studies are demonstrated by the high representation of GO molecular functions comparable with a Human gene database (21,002 entries—Reference Proteomes project at UniProt) (PANTHER) (Fig. 1D). Furthermore, analysis of the B-cell receptor (BCR) signalling pathway (MetaCore, Clarivate, PA, USA) showed that the library incorporates over 87% of the molecules involved in BCR signalling (Supplementary Fig. S1).

### Identification of technical variations in SWATH-MS data

**Figure 2:**
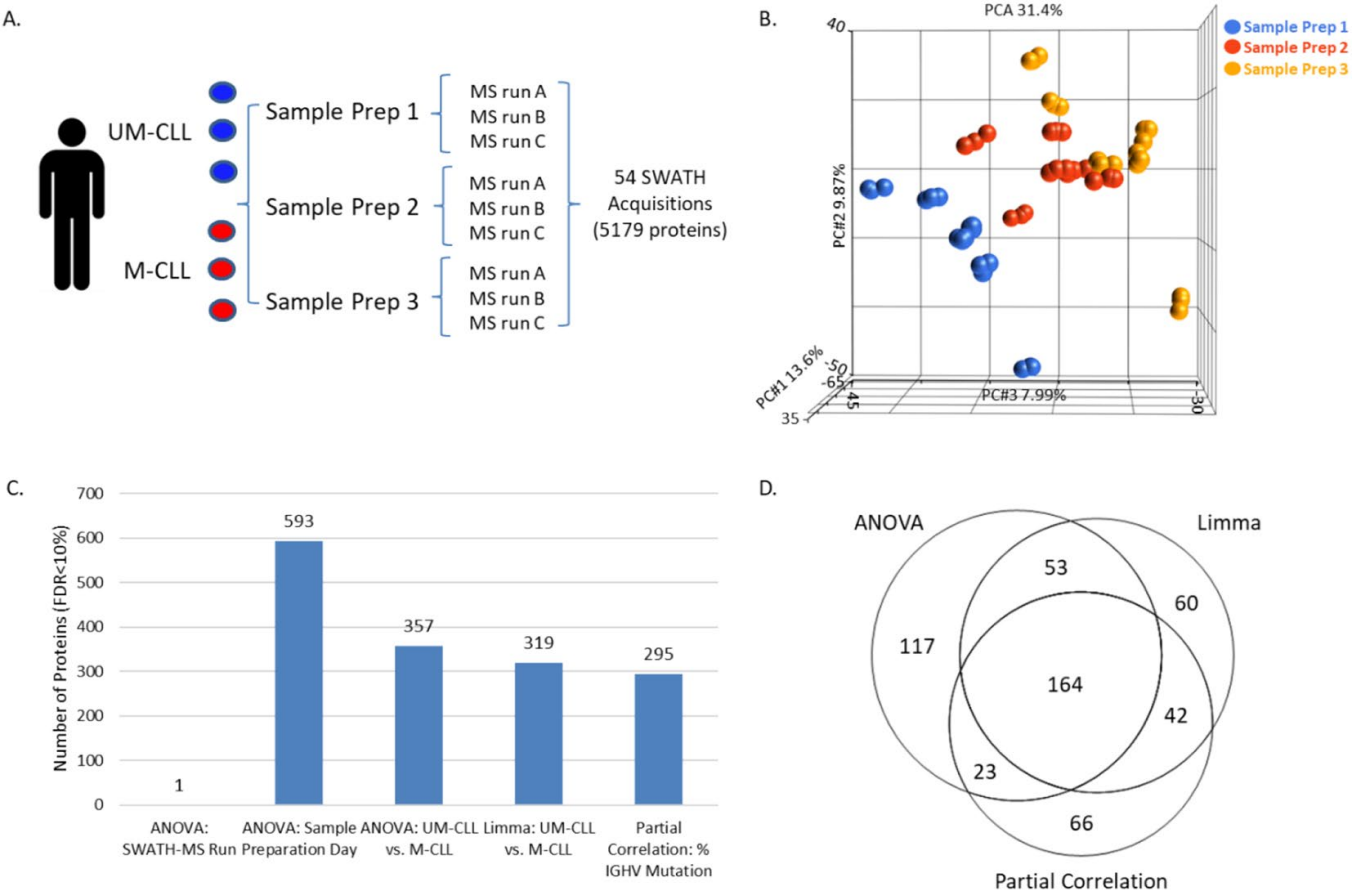
Identification of variation in SWATH-MS data associated with biological and technical factors. (**A**) Experimental design showing 6 CLL patients samples (3 UM-CLL and 3 M-CLL) with replicate sample preparations (n = 3), each analysed in triplicate by SWATH-MS, resulting in 54 acquisitions and quantitative information for 5179 proteins across all samples. (**B**) PCA plot of uncorrected protein expression data showing sample clustering based on sample preparation day. (**C**) Results of ANOVA, limma and partial correlation analysis, highlighting the number of differentially expressed proteins identified (≤ 10% FDR) which are associated with technical variables (SWATH-MS run and sample preparation day) or biological variables (IGHV mutational subgroup (UM-CLL/M-CLL) and % of IGHV mutation). (**D**) Venn diagram showing overlap of proteins found to be differentially expressed between UM-CLL and M-CLL by ANOVA and limma and proteins correlating with the percentage of IGHV mutation by partial correlation analysis.

The biological and technical variability in SWATH data were investigated using cryopreserved CLL samples from 6 patients (Table 1 and Fig. 2A). To ensure biological variability in the samples used in the study, samples with IGHV mutations ranging from 0 to 14% were chosen. Quantitative information was obtained for 5179 proteins and 23,879 peptides across all samples and all replicates by SWATH-MS, which was reduced to 5108 proteins after removing redundancy and weak signals (see “Experimental Procedures”). The full list of proteins is provided in the “Supplementary data”.

**Table 1:** Clinical features of CLL samples analysed by SWATH-MS to identify variation in proteomics data associated with biological and technical factors.

CLL patient	Age at diagnosis	Gender M/F	Prior therapy Y/N	Chromosomal abnormalities	IGHV %	IGHV mutational status	WBC (× 10^9^/l)	WBC class	% Viable cells after thaw (Prep 1, 2, 3)
1	NA	F	NA	NA	4.67	M-CLL	45.8	Low	78%, 71%, 64%
2	77	M	N	13q−	5.42	M-CLL	62.7	Low	81%, 70%, 60%
3	46	M	N	17p−, 13q−	14	M-CLL	359.8	High	87%, 60%, 80%
4	65	M	Y	11q−	0.68	UM-CLL	366.5	High	78%, 79%, 77%
5	58	M	N	12+	0	UM-CLL	34.9	Low	87%, 88%, 84%
6	74	M	N	17p−	0	UM-CLL	57.4	Low	75%, 75%, 83%

IGHV refers to somatic mutation in the IGHV gene of CLL cells compared with the gene sequence of the nearest germ line, where < 2% was classed as UM-CLL and > 2% was classed as M-CLL. WBC refers to the white blood count of the patient at time of sampling. > 300 × 10^9^/l was classed as high WBC and < 100 × 10^9^/l was classed as low WBC in this cohort. Prior therapy consisted of various combinations of glucocorticoid, chlorambucil, fludarabine, or fludarabine plus cyclophosphamide. CLL samples were tested by interphase fluorescence in situ hybridization for del17p13 (17p−), del11q23 (11q−), trisomy 12 (12+, and del13q14 (13q−). 17p and 11q- are regarded as high-risk chromosomal abnormalities.

Overall reproducibility of the SWATH-MS data was initially assessed by performing a PCA. The visual inspection of the samples projected in the first two components revealed that sample preparation was a major source of technical variation, with samples clustering based on preparation day (Fig. 2B and Supplementary Fig. S2A). To identify the number of proteins whose variations were associated with technical factors, the SWATH-MS data were subjected to an ANOVA analysis (Fig. 2C). Replicate SWATH-MS acquisitions exhibited very good reproducibility with minimal technical effects on data. Only a single protein was found to be differentially expressed by ANOVA between the replicate MS acquisitions. In contrast, the replicate sample preparations showed considerable technical variation with 593 proteins found by ANOVA to be differentially expressed between sample preparation days. The ANOVA identified 357 proteins which were differentially expressed between UM-CLL and M-CLL samples.

Two additional computational methods for identifying biologically relevant differences in protein expression were then tested (Fig. 2C). The first was limma. This approach, which is similar to ANOVA, identified 319 proteins associated with IGHV mutational status. The second method was partial correlation, a correlation based approach to identify proteins whose expression correlates with the percentage of IGHV mutation as a continuous variable. Results showed that 295 proteins significantly correlated with the percentage of IGHV mutation. Of these proteins, 187 (63%) had been identified by ANOVA to be differentially expressed between the two IGHV groups, M-CLL and UM-CLL (Fig. 2D).

### Assessments of method to remove batch effects

Having ascertained that the main source of variation was associated with the sample preparation batch, methodologies that correct for this bias were explored. The Bayesian method Combat was chosen to correct for the variation associated with different batches of protein preparation. This method can be used in both supervised (Combat S) and unsupervised (Combat U) modes. Combat S operates with the knowledge of both technical (sample preparation day) and biological factors (IGHV mutational status, WBC and gender) whereas Combat U is only aware of the technical sample groups. In addition, the “RemoveBatchEffect” function from the limma package (limma S) was tested. Limma was also conducted, including both machine run and preparation day into the linear model design (linear M)^18^. After processing the data with the different batch correction methods, ANOVA and limma were used to assess the relative efficacy of the methods to remove technical variation while preserving biological information.

**Figure 3:**
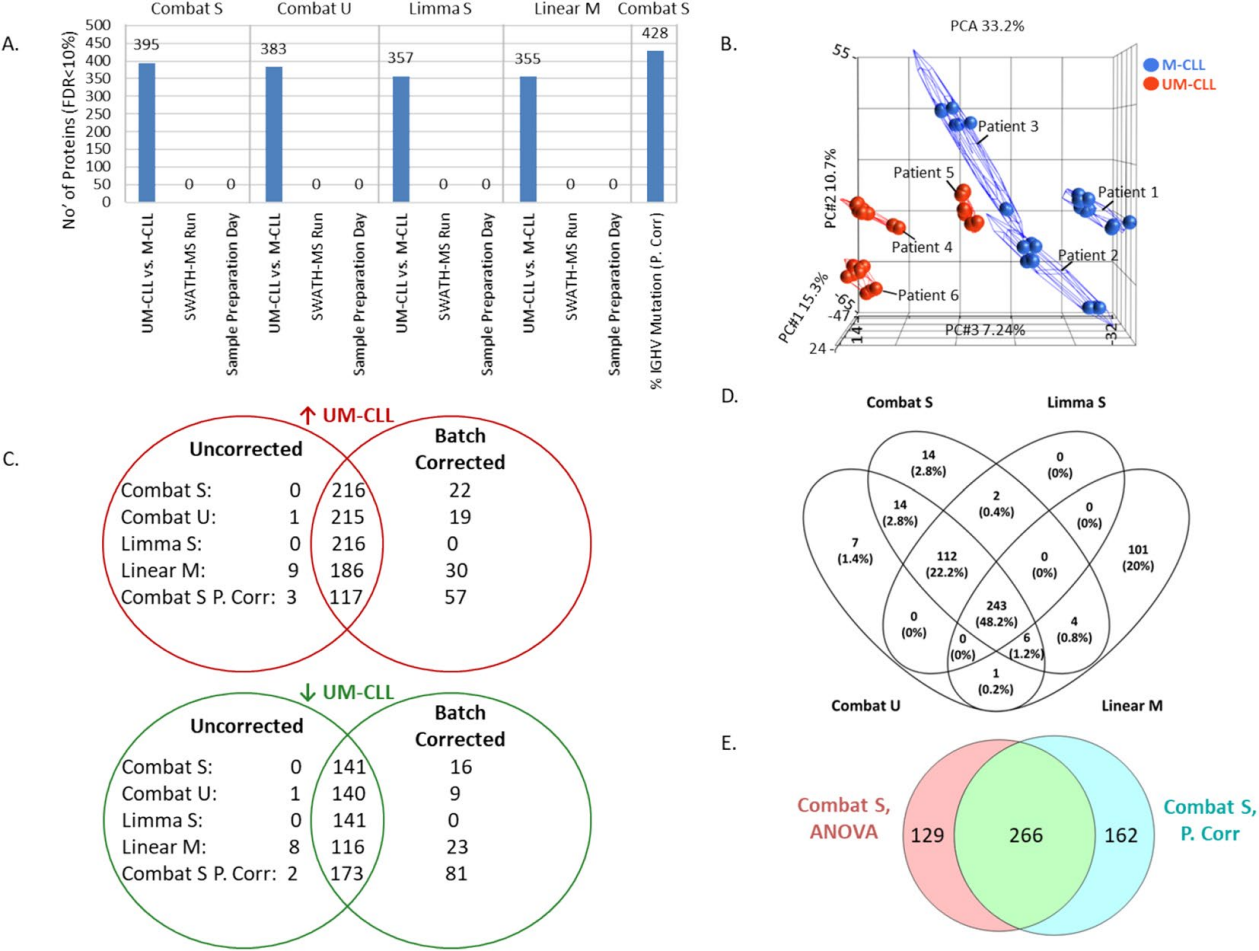
Assessment of methods to remove batch effects in SWATH-MS data. (**A**) Results of ANOVA, limma and partial correlation analysis (P. Corr) on SWATH-MS data that has been batch-corrected using either Combat S, Combat U, limma S or limma with batch information incorporated into the linear model design (linear M) methods. The graph shows the number of differentially expressed proteins (≤ 10% FDR) found to be associated with technical (sample preparation day and SWATH-MS run) or biological (IGHV subgroup (UM-CLL/M-CLL) and % of IGHV mutation) variables. (**B**) PCA plot of SWATH-MS data after processing using Combat S showing clustering based on patient samples (1–6) and on UM-CLL (red) or M-CLL (blue) IGHV mutational status. (**C**) Overlap of proteins found to be significantly associated with IGHV mutation (FDR < 10%) in the SWATH-MS data before (uncorrected) and after batch correction. (**D**) Venn diagram showing overlaps of differentially expressed proteins (UM-CLL/M-CLL) (FDR < 10%) in data which has been batch corrected using Combat S, Combat U, limma or linear M. (**E**) Venn diagram showing overlap of proteins found to be differentially expressed between UM-CLL and M-CLL by ANOVA (FDR < 10%) and significant correlation to percentage of IGHV mutation by partial correlation analysis (P. Corr) (FDR < 10%), after Combat S batch correction.

All of the batch correction methods tested effectively removed variation associated with sample preparation day whilst retaining a comparable number of differentially expressed proteins associated with IGHV mutation status (Fig. 3A). The results were consistent with PCA, which showed that the data now clustered based on patient samples and IGHV mutational status (Fig. 3B).

Both supervised and unsupervised Combat batch correction methods resulted in an increase in the number of proteins found to be significantly differentially expressed between UM-CLL and M-CLL samples, with an additional 26 and 38 proteins identified in the Combat U data and the Combat S data, respectively. Limma S correction showed no difference in the number of differentially expressed proteins identified, whilst the linear M method resulted in an additional 36 significant proteins. Crucially, 100%, 99%, 100% and 95% of the proteins found to be significant to IGHV mutational status in the uncorrected data were retained after Combat S, Combat U, limma S and linear M corrections, respectively (Fig. 3C). Two-hundred and forty three proteins significant to IGHV mutational status were common across all four batch corrected datasets (Fig. 3D). Similar results were observed when analysing the number of proteins found to be significantly differentially expressed between low and high WBC subgroups (Supplementary Fig. S2B). On average, 97% of differentially expressed proteins were retained after batch correction (Supplementary Fig. S2C) and 229 were common across all four batch corrected datasets (Supplementary Fig. S2D).

By far, the largest correction effect was observed on the data analysed by partial correlation, which resulted in an additional 133 proteins significantly associated with the percentage of IGHV mutation after Combat S batch correction (Fig. 3C). Ninety-eight percent of the proteins found to be significant in the uncorrected data were retained after batch correction (Fig. 3C). An overlap of 62% (n = 266) was observed between proteins found to be significant to the percentage of IGHV mutation and proteins found to differentially expressed between M-CLL and UM-CLL samples in the Combat S corrected data (Fig. 3E).

### Analysis of the proteomics IGHV mutational signature identifies functional pathways and upstream regulators in CLL

**Figure 4:**
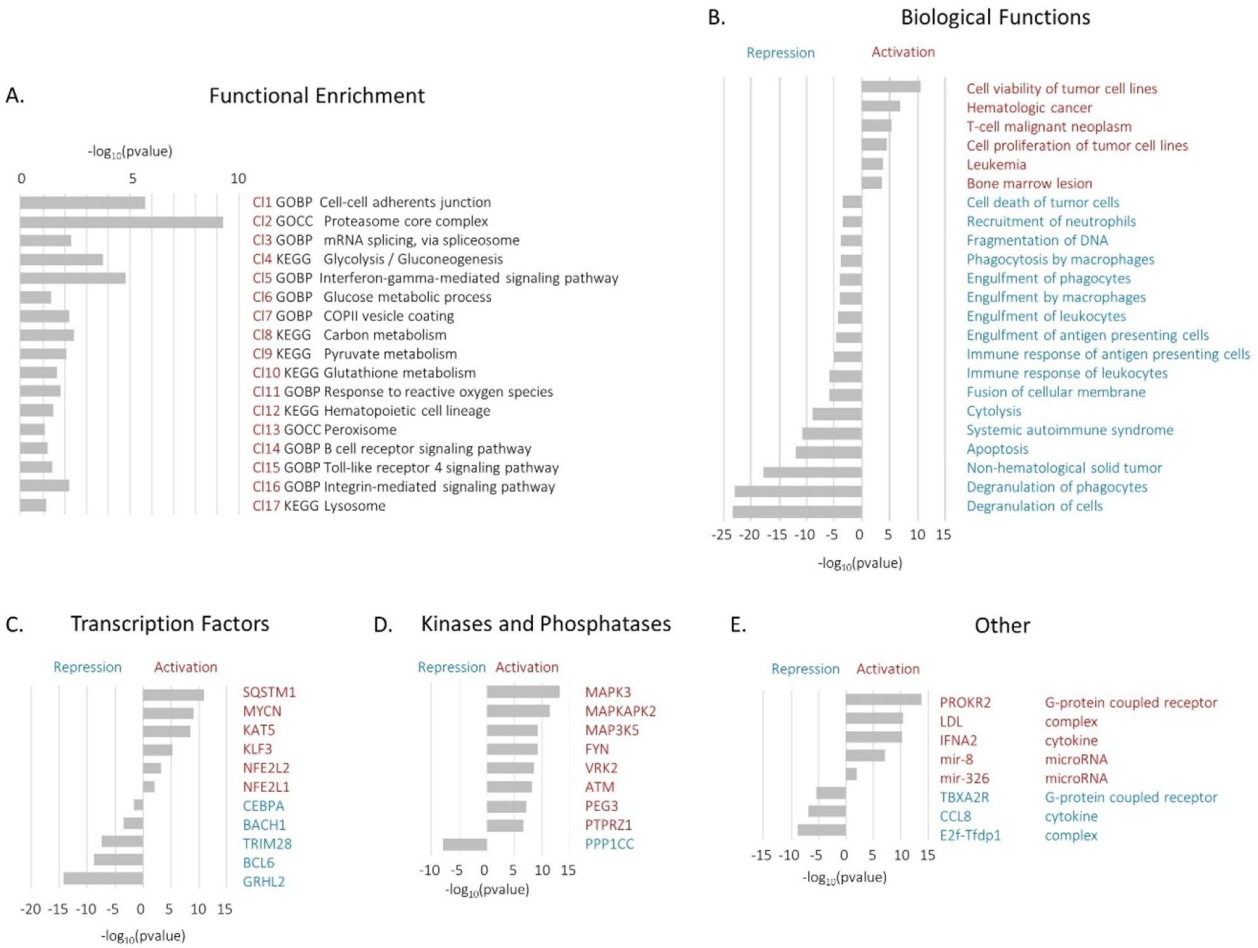
Biological significance of proteins linked to IGHV mutational status. (**A**) Functional annotation analysis performed with the web-based tool DAVID. Representative functional terms for each DAVID cluster have been reported alongside their corrected p value. (**B**) Functional prediction analysis performed using the IPA software. Functional terms predicted to be activated or repressed in UM-CLL compared to M-CLL samples are listed alongside the corrected p value. IPA driver analysis where (**C**) transcription factors, (**D**) kinases and phosphatases and (**E**) other relevant regulatory molecules that are predicted to be activated or repressed in UM-CLL versus M-CLL samples are listed alongside their P values.

To determine the biological significance of proteins found to be differentially expressed between UM-CLL and M-CLL after batch correction, 395 proteins identified by ANOVA after Combat S batch correction were subjected to functional enrichment analysis using a combination of the web-based tool DAVID and Ingenuity Pathway Analysis (IPA). First, DAVID was used to determine whether the list of differentially expressed proteins were enriched in biological pathways. Results showed that significantly enriched pathways included several metabolic functions (Glycolysis, Carbon and Pyruvate metabolism, Glutathione metabolism), adhesion (Cell–cell adherence function), splicing and importantly B cell receptor and Toll-like receptor signalling (Fig. 4A). The IPA software application was then used to infer which functions may be activated or repressed in UM-CLL compared to M-CLL samples. IPA is able to infer a functional response by comparing the observed change in protein expression with prior knowledge of expected effects between regulatory and effector genes stored in the Ingenuity Knowledge database. This approach was applied to identify which biological functions were likely to be activated or repressed, as well as to highlight proteins not detected by the SWATH-MS analysis, which may be responsible for driving the observed differences in the proteomic profile.

The analysis of biological pathways (Fig. 4B) correctly identified the samples as a haematological malignancy and predicted an increase in proliferation and survival and a decrease in apoptosis in UM-CLL cells, an observation consistent with IGHV mutational status^19^. The analysis also predicted an inhibition of phagocytosis which is consistent with a recent observation^20^. The upstream driver analysis inferred changes in the activity of the transcription factors SQSTM1 and GRHL2 (Fig. 4C), the kinases MAPK3 and PPP1CC (Fig. 4D), and the G protein coupled receptor PROKR2 and the E2F Tfdp1 complex (Fig. 4E). These results provide a few intriguing hypotheses on the biology of UM-CLL cells.

### Proteomic and transcriptomic signatures linked to IGHV mutational status significantly overlap

Having shown that proteins linked to IGHV mutational status were present in pathways and biological processes of interest, any correlation between mRNA expression data and SWATH-MS proteomics data was determined. A transcriptional signature linked to IGHV mutational status was first defined by using one of the largest publicly available datasets of mRNA expression profiles for CLL (GEO database, accession number GSE28654)^21^. In total, 3008 mRNA genes were found to be differentially expressed between UM-CLL and M-CLL subgroups (≤ 10% FDR). The mRNA signature was then compared to proteins found to be significant to IGHV mutation in the batch corrected SWATH-MS (FDR ≤ 10%) using GSEA.

Enrichments of all protein sets to the mRNA signature were significant at 0% FDR, with 116, 111, 114 and 118 core genes from the Combat S, Combat U, limma S and linear M corrected SWATH-MS protein data, respectively, overlapping with the mRNA signature (GSEA 0% FDR, Table 2). Interestingly, the protein gene set defined by partial correlation to the percentage of IGHV mutation had the largest core gene overlap with the transcriptional signature, with 139 core genes identified (GSEA 0% FDR, Table 2).

**Table 2:** Normalised enrichment scores and numbers of core genes for the Gene Set Enrichment Analysis (GSEA) of SWATH-MS proteomics data and mRNA expression data.

FC	Protein gene set	Enrichment score	Number of core genes
↑ UM-CLL	Combat S (n = 238)	1.85	80
	Combat U (n = 234)	1.87	78
	limma S (n = 216)	1.88	74
	linear M (216)	2.07	81
↓ UM-CLL	Combat S (n = 157)	− 1.71	36
	Combat U (n = 149)	− 1.65	33
	limma S (n = 141)	− 1.65	40
	linear M (n = 139)	− 1.80	37
Negative P.Corr IGHV	Combat S (n = 174)	2.03	84
Positive P.Corr IGHV	Combat S (n = 254)	− 2.15	55

GSEA was used to compare an mRNA ranked t-statistic gene expression signature (GEO database, accession number GSE28654)^21^, representing genes found to be differentially expressed between UM-CLL and M-CLL samples (≤ 10% FDR, n = 3008), to proteins found to be differentially expressed (≤ 10% FDR) in batch corrected (by Combat supervised (Combat S), Combat unsupervised (Combat U), limma supervised (limma S) or limma with batch information incorporated into the linear model design (linear M)) SWATH-MS data. In addition, the mRNA signature was compared to proteins found to be significantly positively or negatively correlated to the percentage of IGHV mutation by partial correlation analysis (P. Corr) after Combat S batch correction (FDR < 10%). All GSEA results were significant (0% FDR).

### Statistical power analysis

**Figure 5:**
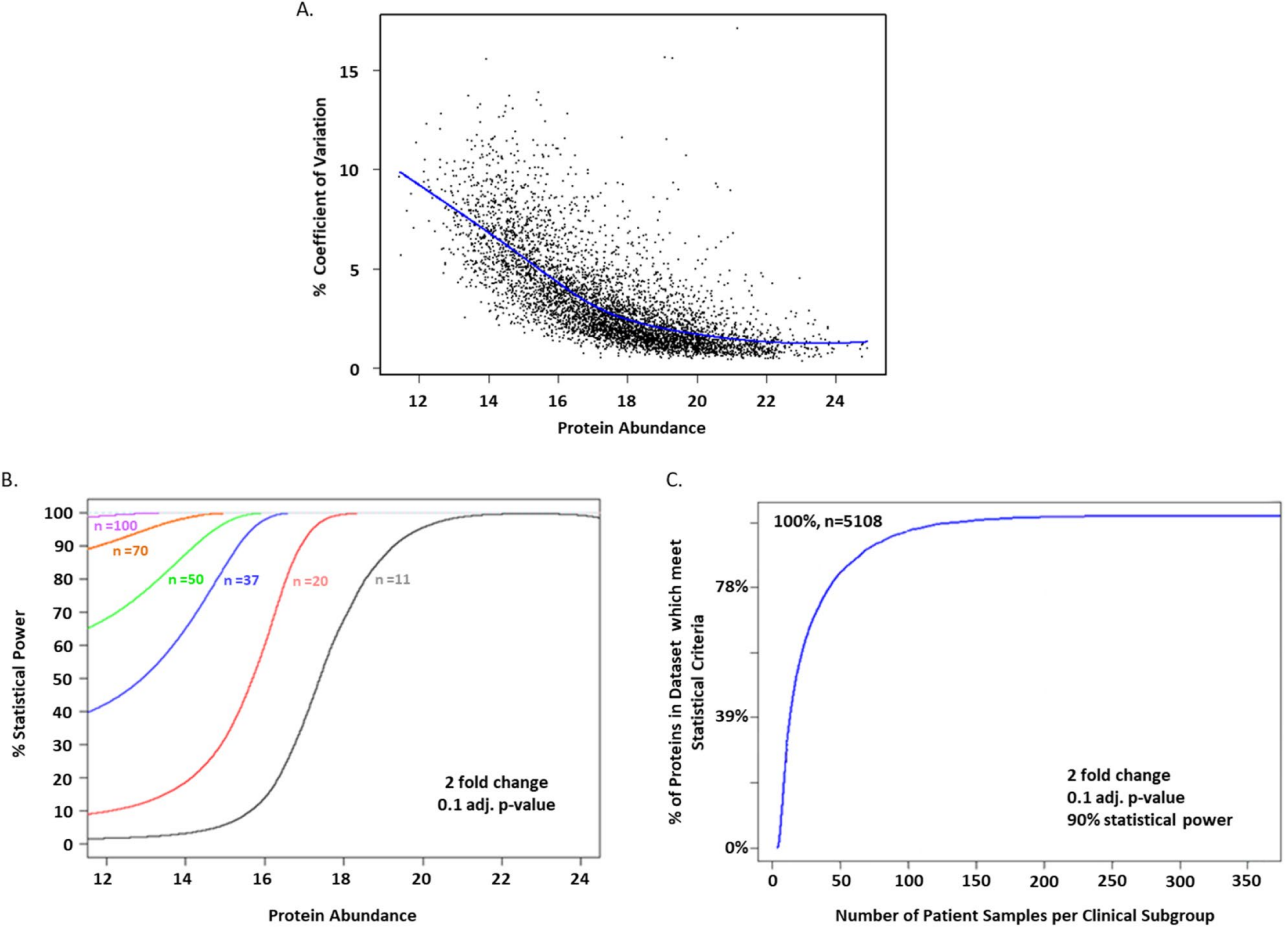
Error model exploration and statistical power analysis of SWATH-MS data. (**A**) Loess model best fit relationship between protein abundance measured by SWATH-MS (total number of proteins = 5108) and coefficient of variation (%). The plot shows that coefficient of variation (%) was higher in proteins expressed at a low abundances. (**B**) Plot of statistical power (%) against protein abundance measured by SWATH-MS (based on the Loess model). The plot shows that to detect a twofold change in protein expression level between clinical subgroups (based on IGHV status) to a Bonferroni corrected alpha of 0.1, both the protein abundance and the number of patient samples per clinical subgroup will influence the statistical power. A higher % statistical power is reached on proteins expressed at low abundances when larger numbers of clinical samples are used. (**C**) Sample size analysis plot based on the SWATH-MS data, showing the number of individuals required per CLL clinical subgroup (IGHV mutational status) to detect a twofold change in protein expression, at 90% Statistical power and to a Bonferroni corrected alpha of 0.1.

A model was built and used to assess the statistical power of CLL SWATH-MS based studies to determine sample sizes suitable for detecting significant changes in protein expression levels between clinical subgroups. Unsurprisingly, the coefficient of variation (%) was dependent upon protein mean abundance, with higher coefficient of variation (%) seen in proteins expressed at low abundances (Fig. 5A).

The relationship between protein abundance and statistical power for a given number of patient samples in each clinical subgroup (i.e. IGHV mutational status) was plotted (Fig. 5B). Visual inspection of this plot shows that even with a small sample size, good statistical power can be achieved across a considerable proportion of the signal range, at least with this dataset. As an example, the percentage of proteins that can be analysed at an estimated 90% statistical power as a function of the sample numbers was plotted (Fig. 5C.). Table 3 shows the percentage of proteins (out of n = 5108 proteins) which would meet the statistical criteria with a given number of patient samples per clinical subgroup if statistical powers of 95%, 75% or 50% are used.

**Table 3:** Required number of patient samples per clinical subgroup and the percentage of proteins in the SWATH-MS dataset which would meet the statistical criteria at a statistical power of 95%, 75% or 50%.

Number of patient samples per clinical subgroup	% of proteins in dataset* which meet statistical criteria		
	95% power	75% power	50% power
5	0	0	0
10	32	45	53
20	57	63	68
30	64	71	80
40	70	79	95
50	76	90	100
75	93	100	100
100	100	100	100

The total dataset consisted of 5108 proteins. The calculations were based on a twofold change and a 0.1 Bonferroni adjusted P-value.

## Discussion

Studies of the proteome are essential if the complexity of disease heterogeneity is to be fully understood, and predictive biomarkers of disease progression and treatment response are to be established. Recently developed DIA-MS methods such as SWATH-MS provide an opportunity to do this, but these are only valid if the variability of the data is recognised and accounted for. Sources of technical variability are numerous and although some procedures can be automated, it is not always possible to remove all aspects of variability in sample preparation. In addition, heterogeneity in diseases such as CLL may be manifested at the protein level, therefore the sample numbers required for SWATH-MS studies must be determined by statistical means in order to reliably detect genuine differential protein expression between clinical subgroups.

Here, a CLL-specific spectral library from CLL patient samples and normal B-cells has been generated. Including normal B-cells in the library not only allows for comparative studies of malignant and normal B-cells in the future, but also captures any differences in the B-cell proteome from very early stages of the disease. The complex mixture of peptides was subjected to extensive fractionation to build the comprehensive library, which captured 50% of all human proteins which have evidence of expression. This is a significant observation given that only a very small proportion of human tissue, i.e. PBMCs, was included. In total, 7736 proteins are represented in the CLL-specific library. In contrast with the CLL proteomics study using iTRAQ-MS published previously by this group^7^, SWATH-MS is a label-free method that allows hundreds of CLL samples to be screened over the course of months or even years. The library provides a permanent reference source that can be readily used by the CLL research community. It is worth mentioning that different peptides are enriched by different sample preparation methods. Therefore, if libraries for SWATH-MS are to be shared, the same sample preparation method used to generate the library should also be used to generate the SWATH maps. To illustrate this point, we have previously prepared CLL samples using hydrophilic interaction liquid chromatography (HILIC) and compared to samples prepared with CEX. SWATH maps from samples prepared by HILIC aligned poorly to the library generated by peptides prepared by CEX. Although 94% of the proteins identified (by DDA) were represented within the CLL spectral library, only 14.5% of peptides were shared between HILIC and CEX prepared samples (data not shown).

SWATH-MS data were acquired from CLL patient samples incorporating triplicate sample preparations from cryopreserved cells and triplicate MS acquisitions. In doing so, effects from cell sample thawing on different dates, batches of chemicals and buffers, changes in analytical columns and maintenance of instruments have been taken into consideration, all of which could contribute to variation in the data. SWATH files were aligned to the CLL spectral library using endogenous CLL peptides in order to calibrate for retention time. Endogenous peptides have been shown to exhibit lower absolute error when compared to spiked-in reference peptides for human lysates^22^. Using this approach, 5179 proteins were quantified across all 54 SWATH maps. In addition to DIA, DDA was also performed on each of the sample preparation replicates. Overlap of DDA and SWATH-MS data was high, with 92% of proteins identified by DDA (< 1% FDR) also identified by SWATH-MS. However, an additional 3152 proteins (156%) were identified by SWATH-MS. Furthermore, on average, only 56% of proteins identified by DDA were common across all three sample preparation replicates, highlighting the problems faced with traditional DDA methods with regards to incomplete datasets (Supplementary Table S1).

Reproducibility between replicate SWATH-MS runs was extremely good (Supplementary Fig. S2A). Although protein variance is reduced during inference of protein abundance^23^, the run-to-run variability in this case is negligible compared to other contributing factors. In contrast, sample preparation replicates showed substantial variability in the data. PCA of uncorrected data showed that samples clustered based on sample preparation day. Four different batch correction methods that are more commonly used for microarray analysis were successfully applied to correct the proteomics data. All methods tested retained the majority of differentially expressed proteins between clinical variables which were identified in the uncorrected data, whilst successfully removing technical variability associated with day of sample preparation. Importantly, PCA of all batch-corrected data showed that data were clustered based on individual patient samples and also according to IGHV mutation status. The number of differentially expressed proteins significant to IGHV mutation increased by 7%, 11%, 11% and 45% after Combat U, Combat S, linear M and Combat S of data analysed by partial correlation, respectively. Comparable percentage increases were also seen for low versus high WBC CLL subgroups, except for linear M analysis in which there was a 2% decrease in the number of differentially expressed proteins identified.

This was a small study designed to assess the best methods to correct for variability in sample processing. Two different approaches were used to assess the success of the approach in terms of revealing expected enrichment of biological functions associated with unmutated IGHV status. Firstly, differentially expressed proteins were functionally annotated and subjected to pathway analysis (IPA). Metabolic functions dominated the results, with 40% of all differentially expressed proteins being associated with metabolic processes and over a third of pathways related to metabolism. These results suggest considerable differences in metabolic activity between UM-CLL and M-CLL cells. Interestingly, proteins found to be differentially expressed between low and high WBC subgroups in the Combat S corrected data showed a similar metabolic functional signature, with 14/38 of the pathways enriched associated with metabolism (Supplementary Fig. S2E). Unlike normal B-cells, CLL cells are known to store lipids and utilise free fatty acids to produce chemical energy^24–26^. Indeed, increased mitochondrial respiration has been associated with poor prognostic indicators such as UM-CLL and advanced clinical stage (based on higher WBC)^27^. Furthermore, high and low metabolic states have been shown to be representative of CLL disease stage^28^. Taken together, this suggests that metabolic adaption is indeed an important factor in the biology and prognosis of CLL. In addition to metabolism, proteins involved in KEGG pathways such as the regulation of actin cytoskeleton and the cell adhesion molecules were also found to be differentially expressed between UM-CLL and M-CLL. These results correlate with our previous CLL iTRAQ-MS study, in which significant differences in cytoskeletal remodelling, cell migration and adhesion pathways were observed between M-CLL and UM-CLL cells^7^.

Pathway analysis provided a few intriguing hypotheses on the biology of UM-CLL cells. It revealed activation of biological functions associated with increased cancer cell survival and repression of those associated with the immune clearance of cancer cells in the UM-CLL samples, in line with previous studies^19,20^. IPA also predicted that the transcription factor SQSTM1 (p62) will be activated in UM-CLL samples (Fig. 4C), promoting nuclear accumulation of NFE2L2/NRF2 and subsequent expression of cytoprotective genes^29,30^. Highly active p62 cells may therefore be more resistant to ROS inducing therapeutics^31^. In addition, IPA predicted an overactivation of the G protein coupled receptor PROKR2, a receptor for prokineticins. These belong to a family of highly conserved small peptides that control a wide range of physiological and pathological functions and which have been implicated in several forms of cancer^32^. Also, prokineticins are expressed at high levels in the bone marrow by monocytic/granulocytic lineage cells^33^. These findings suggest that prokineticins may be relevant in CLL and possibly linked to mutational status. In summary, the functional analysis of protein differences between M-CLL and UM-CLL following stringent batch correction suggest that genuine differences in biology have been captured. It also shows that, despite the relatively small number of samples examined, SWATH-MS analysis has the potential to provide important biological insights.

In the second approach used to validate the SWATH-MS data, GSEA was used to compare a publicly available CLL mRNA expression dataset with the SWATH-MS protein expression datasets. The analysis revealed significant overlaps between differentially expressed transcripts and proteins. Results were consistent across all of the batch correction methods tested, with over 100 core genes identified in each of the five corrected protein expression datasets.

Statistical power analysis was performed using the SWATH-MS data to determine sample sizes suitable for detecting significant changes between clinical subgroups at the protein level. Statistical power analysis with proteomics data is more complex than with traditional data, since the variability between measurements is a function of signal intensity, and statistical power varies between groups of proteins at different levels of expression. Therefore, a model was built and used to assess the statistical power of a CLL SWATH-MS study. Results showed that the number of patients in a clinical subgroup and the protein abundance can affect statistical power. Therefore, to detect significant differences in those proteins expressed at lower levels, larger numbers of clinical samples are required. For example, 100 samples per clinical subgroup would be required to detect significant changes across all proteins in the dataset (n = 5108/5108) with 95% statistical power, whereas 20 samples per group would detect significant changes across 57% of the proteins in the dataset (n = 2912/5108), which would likely be those proteins expressed at higher levels.

This study provides an exhaustive library of CLL proteins, a valuable resource for the research community. It also highlights the critical importance of assessing biological and technical variation in MS data prior to undertaking large-scale, long term proteomic studies of clinical samples. In the case of CLL samples, where the cells have been aliquoted and cryopreserved, we would recommend a minimum of two preparations per patient sample for SWATH-MS. Batch correction methods can then be used to remove technical variability in the data. However, a single SWATH-MS data acquisition for each sample replicate is sufficient. Statistical power analysis has shown that the heterogeneous nature of CLL is manifested, at least in part, at the protein level, making the selection of an adequate number of samples to be included in each clinical subgroup vital for the reliable interpretation of disease-relevant proteomics results. Further work is however needed to fully validate the general applicability of our analytical approach.

## Experimental procedures

### Study design and CLL sample preparation

All samples used for this study were obtained with informed consent and with the approval of the North West 2 Research Ethics Committee–Liverpool Central and stored in the Liverpool Bio-Innovation Hub Biobank (LBIH). All methods were performed in accordance with the relevant guidelines and regulations. Venous blood was drawn from CLL patients into tubes containing sodium heparin at a final concentration of 10 units/1 ml of blood. Mononuclear cells were isolated by centrifugation of blood over Lymphoprep (Axis-Shield PoC AS, Oslo, Norway) within 4 h of sampling and stored at − 150 °C within 2 h of separation. Analysis for recurrent chromosomal abnormalities and IGHV gene mutational analysis was performed as described previously^7,34^.

Cryopreserved peripheral blood mononuclear cells (PBMCs) were thawed at 37 °C, diluted slowly in RPMI 1640 and rested for one hour at 37 °C with 5% CO_2_ to recover after thawing. Cell viability after resting was > 70% for all the cases used in this study, with the exception of three cases which were > 60% (Tables 1 and 4). After washing in ice-cold phosphate-buffered saline (PBS), 2 × 10^7^ cells were lysed by sonication on ice in 50 µL of 7 M urea, 2 M thiourea, 40 mM tris (pH 7.5), 4% CHAPS buffer. Protein concentrations were determined using the 2-D Quant Kit (GE Healthcare, UK). Protein was reduced with 5 mM dithiothreitol (DTT) at 37 °C and alkylated with 0.15 M iodoacetamide (IAA), before diluting with 50 mM ammonium bicarbonate followed by overnight digestion with trypsin (Promega). Peptides were then diluted to 5 mL with 10 mM potassium dihydrogen phosphate/25% acetonitrile (ACN) and acidified to < pH 3 with phosphoric acid prior to cation exchange chromatography.

**Table 4:** Clinical features of CLL samples analysed by data dependant acquisition (DDA) to generate a CLL-specific spectral library for mapping data acquired by SWATH-MS.

Sample	Gender	IGHV %	Chromosomal abnormalities	Prior therapy (Y/N)	WBC (10^9^/l)	Lymphocyte count (10^9^/l)	% viable cells after thaw
CLL-1	F	5.9	13q−	N	63.5	63.4	88
CLL-2	M	IGHV failed to identify single dominant clone 5 different clones identified 0–12	13q−, 11q−	N	21.7	15.5	90
CLL-3	M	NA	17p−	Y	48.1	45	78
CLL-4	M	NA	13q−	Y	24.1	21.9	87
CLL-5	M	2.08	13q−	N	144.3	137	80
CLL-6	M	NA	17p−, 13q−	Y	49.2	44.7	82
CLL-7	M	0	Normal	Y	65.4	63.3	84
CLL-8	M	NA	13q−	Y	110.2	102.9	90
CLL-9	F	0	Normal	Y	52.7	46.7	96
CLL-10	F	NA	13q−	N	149.9	144.8	88
CLL-11	F	0	Normal	Y	31.3	27.9	92
CLL-12	M	0	17p−	Y	291.6	NA	70
CLL-13	M	NA	17p−, 13q−	Y	246.4	NA	77
CLL-14	F	5.9	11q−, 12+	Y	19.3	17.4	90

Prior therapy consisted of various combinations of glucocorticoid, chlorambucil, fludarabine, or fludarabine plus cyclophosphamide. IGHV refers to somatic mutation in the IGHV gene of CLL cells compared with the gene sequence of the nearest germ line, where < 2% was classed as UM-CLL and > 2% was classed as M-CLL. CLL samples were tested by interphase fluorescence in situ hybridization for del17p13 (17p−), del11q23 (11q−), trisomy 12 (12+), and del13q14 (13q−). 17p and 11q− are regarded as high-risk chromosomal abnormalities.

### Data dependent acquisition (DDA) for generation of a CLL-specific spectral library

Cryopreserved PMBCs from 14 CLL patients at different stages of the disease were used to generate a CLL-specific spectral library (Table 4). In addition, normal B-cells were also included, after purification by negative selection using a B-cell isolation kit (Miltenyi Biotech, Bisley, UK) from Buffy coats obtained from the National Blood Service (Liverpool, UK). Cells were lysed and 100 μg of protein from each sample was used to create a representative pool (total 1500 μg) which was prepared as described above. Peptides were fractionated on a polysulfoethyl A strong cation-exchange column (200 × 4.6 mm, 5 μm, 300 Å; Poly LC, Columbia, MD) at 1 mL/min using a gradient from 10 mM potassium dihydrogen phosphate/25% ACN (w/v) to 0.5 M potassium chloride/10 mM potassium dihydrogen phosphate/25% ACN (w/w/v) in 75 min. Fractions of 2 mL were collected and were dried by centrifugation under vacuum (SpeedVac, Eppendorf UK Ltd, Stevenage, UK). Fractions were reconstituted in 1 mL of 0.1% trifluoroacetic acid and were desalted using an mRP Hi Recovery protein column 4.6 × 50 mm (Agilent, Berkshire UK) on an Agilent 1200 HPLC system (Agilent)^7^.

Forty desalted fractions were each reconstituted in 0.1% formic acid and 0.5–1 μg of sample was loaded on-column. Peptides were separated by in-line reversed phase chromatography using a nanoACQUITY UPLC Symmetry C18 Trap Column and an ACQUITY UPLC Peptide BEH C18 nanoACQUITY Column (Waters, UK). Peptides were eluted using a gradient of 2–50% ACN/0.1% formic acid (v/v) over 120 min at a flow rate of 300 nL/min. DDA was performed on a Triple TOF 6600 (SCIEX) in positive ion mode using 25 MS/MS per cycle (2.8 s cycle time) and 30 MS/MS per cycle (1.8 s cycle time) to maximise both spectral quality and coverage, and the combined data were searched using ProteinPilot 5.0 (SCIEX) using the Paragon algorithm (SCIEX). The data were searched against the SwissProt database (Nov 2015, 20,193 human entries) with carbamidomethyl as a fixed modification of cysteine residues and biological modifications allowed. Mass tolerance for precursor and fragment ions was 10 ppm. In order to reduce false positives, a false discovery rate (FDR) of 1% was applied using the reversed database as decoy. This resulted in 7736 proteins being included in the CLL library (PRIDE identifier PXD011330)^35^. This equated to protein, peptide and spectra confidence scores as listed in the DDA “Supplementary data”. In order to align SWATH data with the CLL library, only proteotypic peptides with no modifications were required. To this end, a ‘rapid’ search of the data was performed using ProteinPilot. This resulted in the identification of 7386 proteins at 1% FDR.

Proteins represented in the library were functionally classified using the PANTHER (Protein ANalysis THrough Evolutionary Relationships) classification system (http://pantherdb.org, v12.0)^36,37^ and the GeneGo BCR pathway map in the MetaCore database (Version 6.14 build 61,508; Clarivate, PA, USA) was used to assess molecular coverage within this pathway.

### Data independent acquisition (DIA) (SWATH-MS)

Cryopreserved PBMCs from 6 CLL patients (not used for generating the CLL-specific spectral library) were thawed, lysed and 200 μg of protein from each sample was prepared as described above. Individual digests from samples were loaded onto a prepacked ion exchange column (Bio-Scale Mini Macro-Prep High S, BIO-RAD, UK) in 10 mM potassium dihydrogen phosphate/25% ACN (w/v) and eluted in 0.15 M potassium chloride/10 mM potassium dihydrogen phosphate/25% ACN (w/w/v). Four fractions were collected and dried by centrifugation under vacuum. Fractions were reconstituted in 1 mL of 0.1% trifluoroacetic acid and desalted using an mRP Hi Recovery protein column 4.6 × 50 mm (Agilent) on a 1260 Infinity LC system (Agilent).

Fractions were each reconstituted in 0.1% formic acid and pooled in a total volume of 20 μL. Samples where diluted 1:10 and 5 μL aliquots were delivered into a TripleTOF 6600 mass spectrometer (SCIEX) as described above. SWATH acquisitions were performed using 100 SWATH windows of variable effective isolation width to cover a mass range of 350–1250 m/z (Supplementary Table S2).

Spectra were aligned using SWATH 2.0 in the PeakView v2.2 software (SCIEX) against the CLL-specific spectral library (generated from the search result allowing no modifications) (7386 protein entries). Thirteen endogenous peptides were used for retention time calibration (Supplementary Table S3). Data were processed in PeakView using a XIC extraction window of 8 min and XIC width of 75 ppm. Peak areas from peptides with > 99% confidence and < 1% global false discovery rate were extracted using MarkerView v1.2.1 (SCIEX).

### Experimental design and statistical rationale

This study aimed at assessing the relative contribution of technical and biological factors to the variability observed in a SWATH-MS experiment. The experimental design therefore reflects the need for a suitable compromise between assessing the variability of measurements and constraining the experiment within a reasonable size. Replicate PBMC aliquots from 6 individual CLL patients (Table 1) were thawed, lysed and prepared for SWATH-MS (as described above) on three separate days over a period of 3 months. Patient samples were chosen based on IGHV mutational status, with 3 UM-CLL and 3 M-CLL samples included in the experiment. Triplicate SWATH-MS acquisitions were performed on each replicate sample preparation over a period of one month, incorporating changes in columns and traps and maintenance on the LC and MS systems. In total, 54 SWATH acquisitions were performed.

### Assessing technical and biological variability

SWATH-MS protein expression data was normalised using the total area sums (sum of all peak areas used to compute the scaling factor) normalisation strategy in MarkerView and transformed on a log2 scale. A total of 5108 non-redundant SWATH-MS proteins were defined after converting protein accessions to gene symbols and then removing proteins with a low background signal, imputing missing values with random forest^38^ and collapsing multiple protein accessions to one gene symbol (different translational products reduced to one gene). All 54 samples were treated as biological replicates. An exploratory analysis of the full dataset by principal component analysis (PCA) was then performed using the Partek Genomics Suite (version 7.0).

ANOVA (as implemented in the statistical environment R^39^) was used to assess technical and biological variability. Biological factors included in the model were IGHV mutational status (a cut-off value of 2% was applied to distinguish M-CLL from UM-CLL counterparts^3,4^), white blood count (WBC) (two patients with WBC > 300 × 10^9^/L were categorised as having high WBC and four patients with < 100 × 10^9^/L as low WBC) and patient gender. Technical factors included in the model were the sample preparation day and SWATH-MS acquisition. The p values generated by ANOVA were corrected using the Benjamini and Hochberg method to control for multiple testing^40^. Proteins with a ≤ 10% Benjamini and Hochberg control of FDR ANOVA result were identified as being significantly differentially expressed per variable. Venn diagrams were created using Venny 2.1^41^.

Batch correction was performed using the Bayesian method Combat^42^ and linear models for microarray analysis (limma)^43^. These methods were used as implemented in the Bioconductor packages *sva* (v3.24.0) and *limma* (v3.32.2), respectively. Combat was run in both an unsupervised (no knowledge of IGHV mutational status, WBC or gender (Combat U)) and a supervised manner (knowledge of IGHV mutational status, WBC and gender (Combat S)). The batch correction “removeBatch Effect” function from limma was run in a supervised manner (limma S). In addition, a separate approach was used in which batch information was incorporated into the linear model design (linear M)^18^. This approach also included the duplicate correlation function from limma, in which samples were blocked on the technical replicates^42^. Finally, an alternative approach to identify proteomics signatures associated with IGHV mutation was tested. Instead of subdividing patients in two groups (UM-CLL and M-CLL), the percentage of IGHV mutation was used as a continuous variable and partial correlation on Combat S corrected data was used to identify significant proteins, with WBC and gender as confounding variables. This analysis was performed using the ppcor: Partial and Semi-Partial (Part) Correlation function in R (P. corr, v1.1)^44^. PCA was performed using the Partek Genomics Suite v7.0 to assess variance across the batch corrected sample sets. Proteins with a ≤ 10% FDR result were identified as being significant.

### Functional enrichment analysis

Proteins found to be differentially expressed between UM-CLL and M-CLL in the Combat S corrected data (< 10% FDR by ANOVA) were selected for computational functional analysis. Proteins were functionally classified by Gene Ontology Biological Process (GOBP) and the Kyoto Encyclopaedia of Genes and Genomes (KEGG) pathways using the Database for Annotation, Visualization and Integrated Discovery (DAVID) (v6.8)^45,46^. In addition, functional pathway prediction activity and upstream regulator analysis has been performed on the same list of genes using the Ingenuity Pathway Analysis (IPA, v8.5) software (Qiagen).

### Comparison of differential gene expression in UM-CLL vs. M-CLL subgroups between Protein (SWATH) and mRNA data

A publicly available CLL mRNA dataset acquired from 89 patients with known gender and IGHV mutational status (accession GSE28654)^21^ was used to compare protein and mRNA expression. The Affymetrix data was pre-processed by first selecting probe-sets called present in ≥ 14 samples per IGHV mutational subgroup (MAS5^47^), followed by robust multiarray averaging (RMA) normalisation and finally selecting the most reliable gene probe-sets with the JetSet algorithm^48^, resulting in a final set of 10,953 genes.

Upon PCA of the mRNA data, it was observed that the microarray scan date was a source of technical variation, as clusters of samples based on scan date could be seen (Supplementary Fig. S3A). To remove errors associated with technical variations between scan date batches, data were processed using Combat S batch correction of a 30 sample subset, balanced for IGHV mutational status across scan dates, followed by a limma analysis without scan dates in the model^18^. PCA of the processed mRNA expression data showed IGHV mutational status subgroups separated on the first principal component (Supplementary Fig. S3B).

An mRNA ranked t-statistic gene expression signature was defined and gene set enrichment analysis (GSEA)^49,50^ was used to compare the mRNA expression signature representing genes expressed at higher or lower levels in UM-CLL (≤ 10% FDR) to the corrected SWATH-MS data.

### Statistical power and sample size calculations

One of the objectives of this study was to use the experimental data on the six individual CLL patients, stratified by their IGHV mutational status, to estimate the statistical power associated with a given experimental design. A strategy for estimating statistical power needs to consider that experimental variability is a function of signal intensity and that it is higher for proteins expressed at low levels. Therefore, the coefficient of variation of the available biological replicates was modelled as a function of signal intensity based on the replicate SWATH-MS data. A non-parametric regression method of Loess was used to model the best fit of the coefficient of variation versus the mean protein abundance. Using this model, the statistical power as a function of signal intensity was computed for a given effect and sample size.

Sample size calculations were performed using Combat S processed SWATH proteomics data, balanced for IGHV classes^18^, using the Bioconductor ssize package (which facilitates power analysis calculations and visualization of results when large numbers of gene measurements are involved^51^) (R package version 3.4.0).

## Supplementary Information

Supplementary Information.

## Data Availability

All raw and processed MS data have been deposited to the ProteomeXchange Consortium via the PRIDE partner repository with the dataset identifier PXD011330.

## Abbreviations

ANOVA: Analysis of variance
BCR: B-cell receptor
CEX: Cation exchange chromatography
CLL: Chronic lymphocytic leukemia
Combat S: Combat run in a supervised manner
Combat U: Combat run in an unsupervised manner
DDA: Data dependent acquisition
DIA: Data independent acquisition
FDR: False discovery rate
GO: Gene ontology
GOBP: Gene ontology biological process
GSEA: Gene set enrichment analysis
HILIC: Hydrophilic interaction liquid chromatography
IAA: Iodoacetamide
IGHV: Immunoglobulin heavy chain variable region
iTRAQ: Isobaric tags for relative and absolute quantification
KEGG: Kyoto Encyclopedia of Genes and Genomes pathways
limma: Linear models for microarray analysis
limma S: Limma batch correction function (supervised)
linear M: Limma with batch information incorporated into linear model design
M-CLL: IGHV mutated chronic lymphocytic leukemia
MS/MS: Tandem mass spectrometry
P. corr: Partial and semi-partial (Part) correlation function (R)
PANTHER: Protein analysis through evolutionary relationships
PBMC: Peripheral blood mononuclear cells
PCA: Principal component analysis
SVA: Surrogate variable analysis
SWATH: Sequential windowed acquisition of all theoretical fragments
UM-CLL: IGHV unmutated chronic lymphocytic leukemia
WBC: White blood cell count
