# Supplementary material for: Assessing technical and biological variation in SWATH-MS-based proteomic analysis of chronic lymphocytic leukaemia cells
Source: Sci Rep. 2021 Feb 3;11:2932. doi: 10.1038/s41598-021-82609-2 (PMC7858606; doi:10.1038/s41598-021-82609-2)
Supplement: Supplementary file 1 — Supplementary Information. [file 41598_2021_82609_MOESM1_ESM.pdf]

Assessing Technical and Biological Variation in SWATH-MS-Based Proteomic Analysis of Chronic Lymphocytic Leukaemia Cells

Gina L Eagle, John M J Herbert, Jianguo Zhuang, Melanie Oates, Umair Khan, Neil R Kitteringham, Kim Clarke, B Kevin Park, Andrew R Pettitt, Rosalind E Jenkins, Francesco Falciani.

- Table S1 - Overlap of proteins identified by Data Dependant Acquisition (DDA) in 3 independent sample preparations of 6 CLL patient samples.
- Table S2 – Details of SWATH acquisition windows (n=100) of variable isolation widths.
- Table S3 - Endogenous CLL peptides used for retention time alignment of SWATH maps to the CLL spectral library.
- Fig S1 - Coverage of proteins captured in the CLL spectral library which are Involved in B-Cell Receptor Signalling.
- Fig S2 - Assessment of methods to remove batch effects in SWATH-MS data.
- Fig S3 - Principal Component Analysis of CLL mRNA expression data.

Table S1. Overlap of proteins identified by Data Dependant Acquisition (DDA) in 3 independent sample preparations of 6 CLL patient samples.

CLL Patient	Total No' of Proteins Identified (FDR<1%)	No' Identified Across All 3 Sample Preps	Identified in Sample Preps 1 + 2	Identified in Sample Preps 1 + 3	Identified in Sample Preps 2 + 3
1	2999	1656	1803	1873	1822
2	3017	1705	1835	1936	1859
3	3013	1701	1811	2057	1837
4	3002	1725	1849	1972	1894
5	2856	1578	1678	1743	1863
6	3072	1716	1852	2029	1834

Number of proteins identified (<1% FDR) by DDA from three replicate sample preparations (Sample Prep 1, 2, 3) of 6 CLL patient samples.

Table S2. Details of SWATH acquisition windows (n=100) of variable isolation width (mass range of 350-1250 m/z).

Mass Range (m/z)		Window Size (Da)
349.50	368.60	19.10
367.60	385.30	17.70
384.30	402.70	18.40
401.70	415.80	14.10
414.80	425.90	11.10
424.90	433.90	9.00
432.90	439.70	6.80
438.70	444.80	6.10
443.80	449.10	5.30
448.10	453.10	5.00
451.70	456.70	5.00
455.40	460.40	5.00
459.00	464.00	5.00
462.60	467.60	5.00
466.20	471.20	5.00
469.90	474.90	5.00
473.50	478.50	5.00
477.50	482.50	5.00
481.50	486.50	5.00
485.50	490.50	5.00
489.50	494.50	5.00
493.50	498.50	5.00
497.50	502.50	5.00
501.50	506.50	5.00
505.50	510.50	5.00
509.50	514.50	5.00

513.50	518.50	5.00
517.50	522.50	5.00
521.50	526.50	5.00
525.50	530.50	5.00
529.50	534.50	5.00
533.50	538.50	5.00
537.50	542.50	5.00
541.50	546.50	5.00
545.50	550.50	5.00
549.50	554.50	5.00
552.50	557.90	5.40
556.90	562.20	5.30
561.20	566.20	5.00
564.80	570.20	5.40
569.20	574.50	5.30
573.50	578.90	5.40
577.90	583.20	5.30
582.20	587.60	5.40
586.60	591.90	5.30
590.90	596.30	5.40
595.30	600.60	5.30
599.60	605.00	5.40
604.00	609.30	5.30
608.30	613.70	5.40
612.70	617.70	5.00
616.30	621.70	5.40
620.70	626.00	5.30
625.00	631.10	6.10
630.10	635.40	5.30
634.40	639.80	5.40
638.80	644.90	6.10

643.90	649.90	6.00
648.90	655.00	6.10
654.00	660.10	6.10
659.10	665.90	6.80
664.90	671.00	6.10
670.00	676.80	6.80
675.80	682.60	6.80
681.60	688.40	6.80
687.40	694.20	6.80
693.20	700.00	6.80
699.00	705.80	6.80
704.80	712.30	7.50
711.30	719.50	8.20
718.50	726.80	8.30
725.80	734.00	8.20
733.00	741.30	8.30
740.30	749.30	9.00
748.30	757.20	8.90
756.20	765.90	9.70
764.90	775.40	10.50
774.40	784.10	9.70
783.10	794.20	11.10
793.20	805.10	11.90
804.10	816.70	12.60
815.70	828.30	12.60
827.30	841.30	14.00
840.30	855.10	14.80
854.10	869.60	15.50
868.60	885.60	17.00
884.60	903.70	19.10
902.70	922.50	19.80

921.50	944.30	22.80
943.30	968.90	25.60
967.90	998.70	30.80
997.70	1036.40	38.70
1035.40	1088.60	53.20
1087.60	1154.50	66.90
1153.50	1249.50	96.00
1248.50	1253.50	5.00

Table S3. Endogenous CLL peptides used for retention time alignment of SWATH maps to the CLL spectral library.

	Protein	Accession	Gene Code	Peptide	RT
1	Talin-1	Q9Y490	TLN1	AVTQALNR	27.78
2	Filamin-A	P21333	FLNA	AFGPGLQGGSAGSPAR	37.25
3	Talin-1	Q9Y490	TLN1	ALSTDPAAPNLK	39.47
4	DNA-dependent protein kinase catalytic subunit	P78527	PRKDC	VTELALTASDR	40.43
5	U5 small nuclear ribonucleoprotein 200 kDa helicase	O75643	U520	IVALSSSLSNAK	44.02
6	Chromodomain-helicase-DNA-binding protein 4	Q14839	CHD4	VGGNIEVLGFNAR	57.1
7	Ras GTPase-activating-like protein IQGAP1	P46940	IQGA1	LGLAPQIQDLYGK	62.5
8	Myosin-9	P35579	MYH9	NFINNPLAQADWAAK	66.72
9	Clathrin heavy chain 1	Q00610	CLH1	ISGETIFVTAPHEATAGIIGVNR	67.68
10	Clathrin heavy chain 1	Q00610	CLH1	SVNESLNNLFITEEDYQALR	73.95
11	DNA-dependent protein kinase catalytic subunit	P78527	PRKDC	AALSALESFLK	76.75
12	Ras GTPase-activating-like protein IQGAP1	P46940	IQGA1	ILAIGLINEALDEGDAQK	81.52
13	DNA-dependent protein kinase catalytic subunit	P78527	PRKDC	LSDFNDITNMLLLK	85.52

Thirteen endogenous peptides were used for retention time calibration and were chosen based on presence in CLL patient samples tested (n>50) good distribution cross chromatogram, high intensity of ionised peptide and absence of modifications.

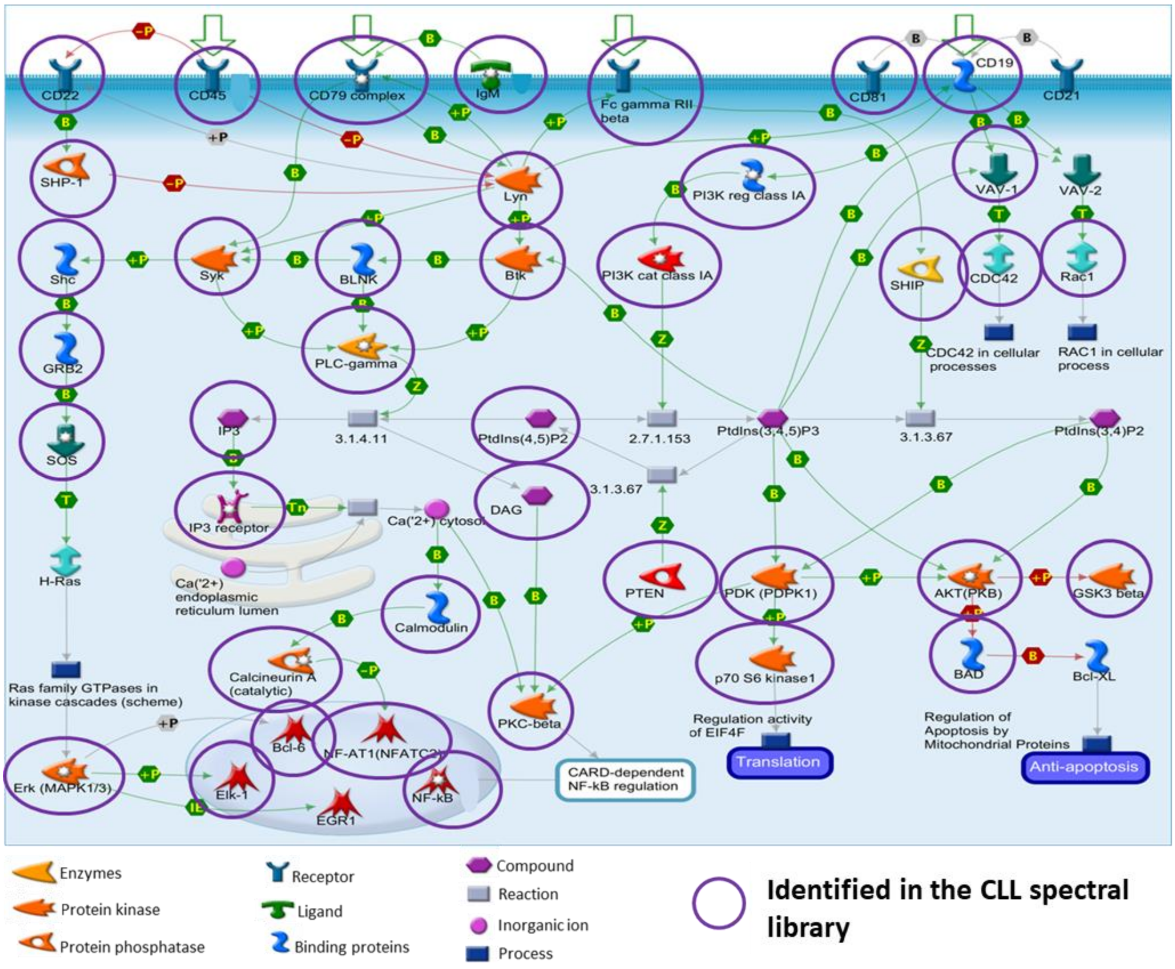

Fig. S1. Coverage of proteins captured in the CLL spectral library which are Involved in B-Cell Receptor Signalling. GeneGo Pathway map of B-Cell Receptor (BCR) signalling in the Metacore database (Version 6.14 build 61508; Clarivate, PA, USA), highlighting the proteins in the pathway which are represented in the CLL spectral library. MetaCore and Clarivate are trademarks of their respective owners and used herein with permission.

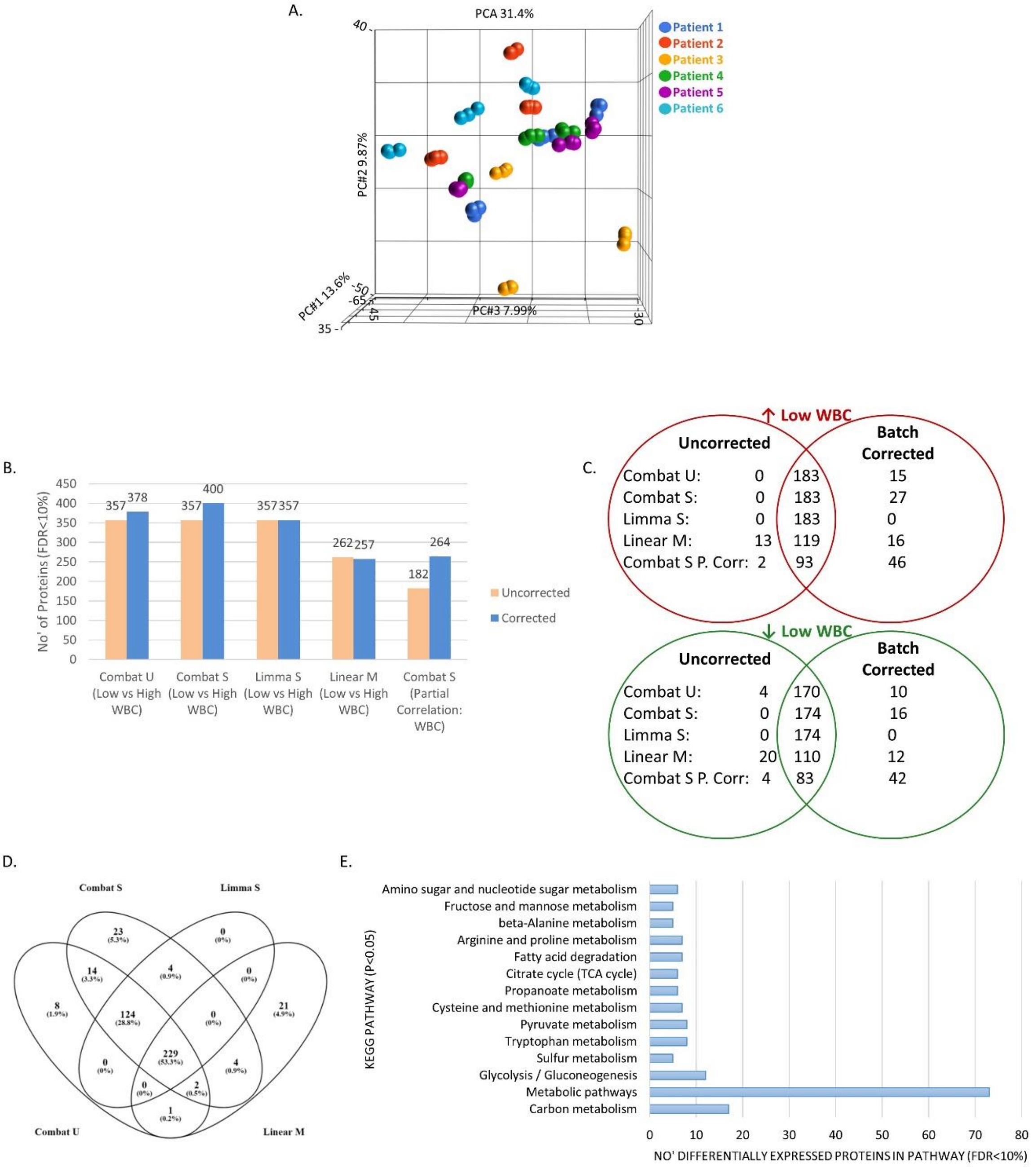

Fig. S2. Assessment of methods to remove batch effects in SWATH-MS data. (A) PCA plot of uncorrected protein expression data. This plot is a recolour of Figure 2B and highlights patient sample. The plot also emphasises the reproducibility of SWATH-MS acquisition, with the triplicate MS runs of each sample from each sample preparation day closely aligned. (B) The graph shows the number of differentially expressed proteins (≤10% FDR) found to be associated with patient white blood count (WBC) by ANOVA, limma and partial correlation analysis on uncorrected SWATH-MS data and after data processing by Combat supervised (Combat S), Combat unsupervised (Combat U), limma

supervised (limma S) or limma with batch information incorporated into the linear model design (linear M) batch correction methods. (C) Overlap of proteins found to be significant with WBC (FDR<10%) in the SWATH-MS data before (uncorrected) and after batch correction. (D) Venn diagram showing overlaps of differentially expressed proteins (low/high WBC) (FDR<10%) in data which has been batch corrected using Combat S, Combat U, limma or linear M. (E) Proteins identified to be differentially expressed between low and high WBC samples by ANOVA in the Combat S corrected data (FDR<10%, n=400) were subjected to functional enrichment analysis using the web based tool, DAVID. The graphs shows KEGG pathways associated with metabolism which were significantly enriched (P<0.05, n=14/38).

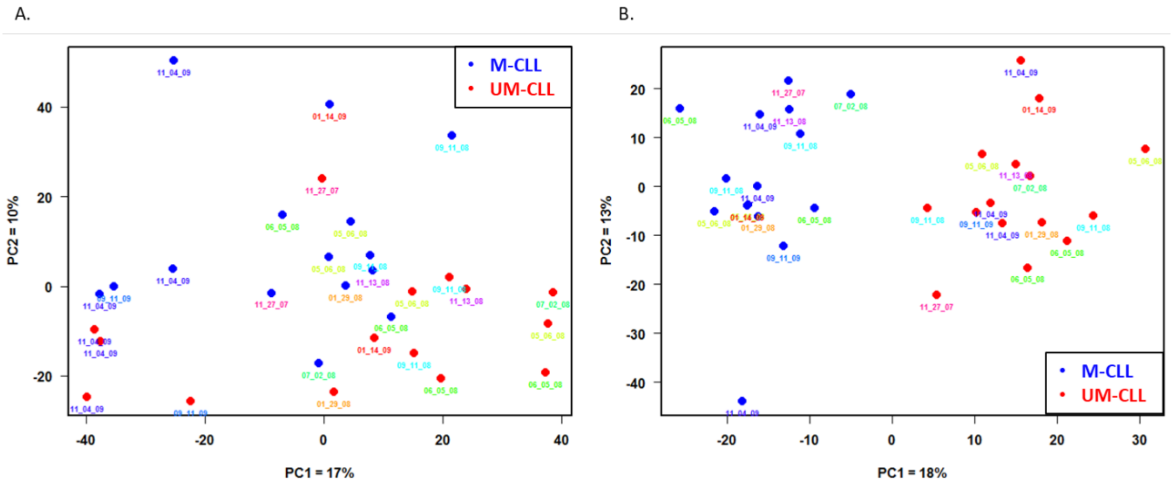

Fig. S3. Principal Component Analysis of CLL mRNA expression data. Principal Component Analysis (PCA) of mRNA expression data from 30/89 CLL samples with scan dates balanced for mutational status (accession GSE28654) (Trojani et al. 2011). (A) Uncorrected mRNA expression data showing no separation of UM-CLL (red) and M-CLL (blue) samples. Scan dates are coloured to show potential batch effects. (B) PCA plot after Combat supervised correction showing separation on the first component of samples based on UM-CLL (red) and M-CLL (blue) mutational status.
